# Safety and efficacy of ruxolitinib cream for the treatment of vitiligo: A randomised controlled trial secondary analysis at 3 years

**DOI:** 10.1002/ski2.404

**Published:** 2024-08-07

**Authors:** John E. Harris, Amit G. Pandya, Mark Lebwohl, Iltefat H. Hamzavi, Pearl Grimes, Alice B. Gottlieb, Howard L. Sofen, Angela Y. Moore, Mingyue Wang, Deanna Kornacki, Kathleen Butler, David Rosmarin

**Affiliations:** ^1^ University of Massachusetts Chan Medical School Worcester Massachusetts USA; ^2^ Palo Alto Foundation Medical Group Sunnyvale California USA; ^3^ University of Texas Southwestern Medical Center Dallas Texas USA; ^4^ Icahn School of Medicine at Mount Sinai New York New York USA; ^5^ Henry Ford Medical Center Detroit Michigan USA; ^6^ The Vitiligo & Pigmentation Institute of Southern California Los Angeles California USA; ^7^ David Geffen UCLA School of Medicine Los Angeles California USA; ^8^ Arlington Research Center Arlington Texas USA; ^9^ Baylor University Medical Center Dallas Texas USA; ^10^ Incyte Corporation Wilmington Delaware USA; ^11^ Indiana University School of Medicine Indianapolis Indiana USA

## ETHICS STATEMENT

The protocol was approved by the institutional review board at each participating site. The study was conducted in accordance with the International Council for Harmonisation guidelines for Good Clinical Practice and the Declaration of Helsinki. All patients provided written informed consent.

## PATIENT CONSENT

Written patient consent for publication was obtained.

## PREVIOUS PRESENTATION

These data have been previously presented in part at the 102nd Annual Meeting of the British Association of Dermatologists (Glasgow, UK; July 5–7, 2022).

Dear Editor,

Vitiligo is a chronic autoimmune disease characterised by destruction of melanocytes resulting in patches of skin depigmentation^1^ that is associated with considerable disease burden.^2^, ^3^ Ruxolitinib (Janus kinase 1/2 inhibitor) cream^4^ demonstrated substantial repigmentation over 52 weeks and was well tolerated in a phase 2, dose‐ranging, randomised study (NCT03099304) in adults (aged 18–75 years) with depigmentation of ≥0.5% and ≥3% of facial and nonfacial body surface area, respectively.^5^ Importantly, repigmentation was sustained over 104 weeks.^6^ We report additional safety and efficacy data from the study following 156 weeks of treatment and provide evidence of treatment durability 6 months after stopping treatment.

Detailed study methodology for the double‐blind, vehicle‐controlled (through Week 24) and double‐blind extension (through Week 52) periods has been published.^5^ After Week 52, all patients could apply 1.5% ruxolitinib cream twice daily (BID) during a 104‐week open‐label extension (OLE; Weeks 52–156), with 3–6 months' follow‐up. During the OLE, patients with complete facial repigmentation could stop treatment or decrease application frequency based on investigator judgement; treatment could be restarted or application frequency increased if pigmentation was lost. Efficacy assessments included the percentage of patients achieving the following at Week 156: facial Vitiligo Area Scoring Index (F‐VASI) improvements of ≥50%/≥75%/≥90% (F‐VASI50/F‐VASI75/F‐VASI90); total Vitiligo Area Scoring Index (T‐VASI) improvements of ≥50%/≥75% (T‐VASI50/T‐VASI75); facial Physician's Global Vitiligo Assessment (F‐PhGVA) response (clear or almost clear skin); and Patient Global Impression of Change–Vitiligo (PaGIC‐V) response (very much or much improved). Adverse event (AE) frequency and haematologic parameters were monitored.

Of 77 patients initially randomised to the three highest doses of ruxolitinib cream (0.5% or 1.5% once daily, or 1.5% BID) who continued in the OLE, mean (SD) age was 51.2 (12.0) years, 57.1% (*n* = 44/77) were male, 87.0% (*n* = 67/77) were White, and 75.3% (*n* = 58/77) had Fitzpatrick skin types I–III. Baseline mean (SD) T‐VASI and F‐VASI scores were 18.7 (16.1) and 1.29 (0.78), respectively. The distribution of baseline disease characteristics was similar among patients initially randomised to 1.5% ruxolitinib cream BID and those continuing in the OLE.

Twenty‐five patients had Week 156 data. Among these, F‐VASI50, F‐VASI75, and F‐VASI90 responses were achieved by 92.0% (*n* = 23/25), 68.0% (*n* = 17/25), and 48.0% (*n* = 12/25) of patients, respectively; T‐VASI50 and T‐VASI75 responses were achieved by 60.0% (*n* = 15/25) and 20.0% (*n* = 5/25), respectively. Additionally, F‐PhGVA and PaGIC‐V responses were achieved by 56.0% (*n* = 14/25) and 64.0% (*n* = 16/25), respectively. Across assessments, responses increased from Weeks 24 through 156; the most rapid improvements and the majority of responses were observed in the first 2 years (Table 1). These findings were consistent among the 33 patients randomised to 1.5% ruxolitinib cream BID on Day 1.

**TABLE 1:** Efficacy of ruxolitinib cream through 3 years of treatment and post‐treatment maintenance of response.

Endpoint, *n*/*N* (%)	Week 24	Week 52	Week 104	Week 156	Maintenance of response^a^
All patients initially randomised to the 3 highest strengths of ruxolitinib cream^b^					
F‐VASI50^c^	38/94 (40.4)	46/94 (48.9)	47/56 (83.9)	23/25 (92.0)	25/31 (80.6)
F‐VASI75^c^	20/94 (21.3)	34/94 (36.2)	37/56 (66.1)	17/25 (68.0)	17/23 (73.9)
F‐VASI90^c^	11/94 (11.7)	20/94 (21.3)	30/56 (53.6)	12/25 (48.0)	11/17 (64.7)
T‐VASI50^c^	13/94 (13.8)	29/94 (30.9)	32/56 (57.1)	15/25 (60.0)	–
T‐VASI75^c^	2/94 (2.1)	9/94 (9.6)	15/56 (26.8)	5/25 (20.0)	–
F‐PhGVA of clear or almost clear^d^	10/94 (10.6)	16/79 (20.3)	24/56 (42.9)	14/25 (56.0)	–
PaGIC‐V of very much or much improved^e^	23/87 (26.4)	33/79 (41.8)	31/56 (55.4)	16/25 (64.0)	–
Patients initially randomised to 1.5% ruxolitinib cream BID					
F‐VASI50^c^	15/33 (45.5)	19/33 (57.6)	17/19 (89.5)	9/9 (100)	–
F‐VASI75^c^	10/33 (30.3)	17/33 (51.5)	14/19 (73.7)	8/9 (88.9)	–
F‐VASI90^c^	4/33 (12.1)	11/33 (33.3)	11/19 (57.9)	6/9 (66.7)	–
T‐VASI50^c^	4/33 (12.1)	12/33 (36.4)	12/19 (63.2)	7/9 (77.8)	–
T‐VASI75^c^	1/33 (3.0)	4/33 (12.1)	5/19 (26.3)	3/9 (33.3)	–
F‐PhGVA of clear or almost clear^d^	3/33 (9.1)	7/29 (24.1)	9/19 (47.4)	8/9 (88.9)	–
PaGIC‐V of very much or much improved^e^	9/31 (29.0)	13/29 (44.8)	11/19 (57.9)	8/9 (88.9)	–
F‐VASI response maintenance at 6‐month follow‐up after stopping treatment^f^					
F‐VASI50–<75	–	–	–	–	6/8 (75.0)
F‐VASI75–<90	–	–	–	–	6/6 (100)
F‐VASI90	–	–	–	–	11/17 (64.7)

Abbreviations: BID, twice daily; F‐PhGVA, facial Physician's Global Vitiligo Assessment; F‐VASI, facial Vitiligo Area Scoring Index; F‐VASI50/75/90, ≥50%/≥75%/≥90% improvement in F‐VASI; PaGIC‐V, Patient Global Impression of Change–Vitiligo; QD, once daily; T‐VASI, total Vitiligo Area Scoring Index; T‐VASI50/75, ≥50%/≥75% improvement in T‐VASI.

^a^ Measured 6 months after stopping treatment in patients who had >52 weeks 1.5% ruxolitinib cream BID treatment and ≥1 nonmissing response value during the follow‐up period after treatment cessation.

^b^ Ruxolitinib cream strengths 0.5% QD, 1.5% QD, and 1.5% BID; all patients applied 1.5% ruxolitinib cream BID after Week 52.

^c^ Missing postbaseline values were imputed as nonresponders up to Week 52; after Week 52, data were reported as observed.

^d^ Missing postbaseline values were imputed as nonresponders up to Week 24; after Week 24, data were reported as observed.

^e^ Data were reported as observed at all time points.

^f^ Only includes patients with nonmissing evaluations at the 6‐month follow‐up.

Repigmentation of facial lesions was maintained for up to 6 months after stopping treatment in more than half of the small number of evaluable patients who applied ruxolitinib cream for >52 weeks (Table 1). Although one‐third of patients were not evaluable at the 6‐month follow‐up visit after stopping treatment, F‐VASI50 and F‐VASI75 were maintained in 75% (*n* = 6/8) and 100% (*n* = 6/6) of evaluable patients, respectively; F‐VASI90 was maintained in 64.7% (*n* = 11/17).

Ruxolitinib cream was well tolerated over 3 years, with no significant safety signals observed or treatment‐related serious AEs reported during the 104‐week OLE, and no accumulation of AEs observed over 156 weeks. Treatment‐emergent AEs occurred in 57.1% (*n* = 44/77) of patients in the OLE, the most common being sinusitis (5.2%, *n* = 4/77). The most common AE among patients who applied 1.5% ruxolitinib cream BID across the entire 3‐year study was acne (18.2%, *n* = 6/33; all grade 1). During the OLE, one serious TEAE (grade 3 acute cholecystitis) was reported in a patient applying 1.5% ruxolitinib cream BID from Day 1, which was considered non–treatment‐related by investigators. In total, 9.1% (*n* = 7/77) had treatment‐related AEs (all grades 1–2). No clinically relevant changes in haemoglobin or platelet levels were observed over 156 weeks, regardless of ruxolitinib cream strength at initial randomisation.

These results add to previously reported efficacy and safety data with ruxolitinib cream, which demonstrated substantial and continuous repigmentation of vitiligo lesions as well as consistent safety profiles after treatment of up to 1 year in both phase 2 and 3 studies.^5^, ^7^ We conclude that ruxolitinib cream produced substantial facial and total body repigmentation of vitiligo lesions and was well tolerated through 3 years of treatment. Facial lesion repigmentation improvements of ≥50% were maintained for up to 6 months among approximately three‐quarters of evaluable patients after stopping treatment. Larger studies are needed to confirm these findings; nonetheless, these data support the long‐term safety and efficacy of ruxolitinib cream for the treatment of vitiligo.

## CONFLICT OF INTEREST STATEMENT

JEH has served as a consultant for AbbVie, Aclaris Therapeutics, BiologicsMD, EMD Serono, Genzyme/Sanofi, Janssen, Pfizer, Rheos Medicines, Sun Pharmaceuticals, TeVido BioDevices, The Expert Institute, 3rd Rock Ventures, and Villaris Therapeutics; has served as an investigator for Aclaris Therapeutics, Celgene, Dermira, EMD Serono, Genzyme/Sanofi, Incyte Corporation, LEO Pharma, Pfizer, Rheos Medicines, Stiefel/GlaxoSmithKline, Sun Pharmaceuticals, TeVido BioDevices, and Villaris Therapeutics; holds equity in Aldena Therapeutics, NIRA Biosciences, Rheos Medicines, TeVido BioDevices, and Villaris Therapeutics; is a scientific founder of Aldena Therapeutics, NIRA Biosciences, and Villaris Therapeutics; and has patents pending for IL‐15 blockade for treatment of vitiligo, JAK inhibition with light therapy for vitiligo, and CXCR3 antibody depletion for treatment of vitiligo. AGP has served as an investigator for Aclaris Therapeutics, Immune Tolerance Network, Incyte, and Pfizer; a consultant for AbbVie, Arcutis, Avita Medical, Chromaderm, Immune Tolerance Network, Incyte, Pfizer, TWi, Viela Bio, and Villaris; and holds stock options for Tara Medical and Zerigo Health. ML is an employee of Mount Sinai Hospital, which receives research funds from AbbVie, Amgen, Arcutis, Avotres, Boehringer Ingelheim, Dermavant, Eli Lilly, Incyte, Janssen Research & Development, LLC, Ortho Dermatologics, Regeneron, and UCB, Inc; and is a consultant for Aditum Bio, Almirall, AnaptysBio, Arcutis, Aristea, Arrive Technology, Avotres Therapeutics, BioMX, Boehringer Ingelheim, Bristol Myers Squibb, Cara Therapeutics, Castle Biosciences, Corrona, Dermavant Sciences, Dr. Reddy's Laboratories, Evelo, Evommune, Facilitate International Dermatologic Education, Forte, Foundation for Research and Education in Dermatology, Helsinn, LEO Pharma, Meiji Seika Pharma, Mindera, Pfizer, and Verrica. IHH has served as an advisory board member for AbbVie; a consultant for Boehringer Ingelheim, Galderma Laboratories LP, Incyte, Pfizer, and UCB; a principal investigator for Avita, Bayer, Estée Lauder, Ferndale Laboratories, Incyte, Lenicura, L’Oréal, Pfizer, and Unigen; immediate past president of the HS Foundation; and a board member of the Global Vitiligo Foundation. PG has served as a consultant for Aclaris Therapeutics, Clarify Medical, DermaForce, Incyte, Proctor & Gamble, and Versicolor Technologies and a principal investigator for Aclaris Therapeutics, Allergan/SkinMedica, Clinuvel Pharmaceuticals, Incyte, Johnson & Johnson, L’Oreal, Merz Pharma, Pfizer, Thync Global Inc., and VT Cosmetics. ABG has received honoraria as an advisory board member and consultant for Amgen, AnaptysBio, Avotres Therapeutics, Boehringer Ingelheim, Bristol Myers Squibb, DICE Therapeutics, Eli Lilly, Janssen, Novartis, Sanofi, UCB Pharma, and Xbiotech, and has received research/educational grants from AnaptysBio, Moonlake IMmunotherapeutics, Novartis, Bristol Myers Squibb, and UCB Pharma (all paid to Mount Sinai School of Medicine). HLS has served as a consultant and/or investigator for AbbVie, Amgen, Asana, Boehringer Ingelheim, Celgene, Dermavant, Dermira, Eli Lilly and Company, Incyte, Janssen/Johnson & Johnson, Kiniksa, LEO Pharma, Sun Pharmaceuticals, UCB, and Xbiotech. AYM has received research funds and/or honoraria from AbbVie, Aclaris, Almirall, Arcutis, Biofrontera, Boehringer Ingelheim, Bristol Myers Squibb, Dermavant, Eli Lilly, Evolus, Galderma, Incyte, Janssen, Johnson & Johnson, Parexel, Pfizer, Takeda, UCB, Verrica, and Vyne. DK is an employee and shareholder of Incyte. MW and KB were employees and shareholders of Incyte at the time of the study. DR has received honoraria as a consultant and/or speaker for and/or received research support from AbbVie, Abcuro, AltruBio, Amgen, Arena, Boehringer‐Ingelheim, Bristol Myers Squibb, Celgene, Concert, CSL Behring, Dermavant, Dermira, Galderma, Incyte Corporation, Janssen, Kyowa Kirin, Lilly, Merck, Nektar, Novartis, Pfizer, RAPT, Regeneron Pharmaceuticals, Recludix, Revolo Biotherapeutics, Sanofi, Sun Pharmaceuticals, UCB, Viela Bio, and Zura Bio.

## AUTHOR CONTRIBUTIONS

**John E. Harris**: Conceptualization (equal); data curation (equal); formal analysis (equal); investigation (equal); methodology (equal); writing – review & editing (equal). **Amit G. Pandya**: Conceptualization (equal); data curation (equal); formal analysis (equal); investigation (equal); methodology (equal); writing – review & editing (equal). **Mark Lebwohl**: Conceptualization (equal); data curation (equal); formal analysis (equal); investigation (equal); methodology (equal); writing – review & editing (equal). **Iltefat H. Hamzavi**: Conceptualization (equal); data curation (equal); formal analysis (equal); investigation (equal); methodology (equal); writing – review & editing (equal). **Pearl Grimes**: Conceptualization (equal); data curation (equal); formal analysis (equal); investigation (equal); methodology (equal); writing – review & editing (equal). **Alice B. Gottlieb**: Conceptualization (equal); data curation (equal); formal analysis (equal); investigation (equal); methodology (equal); writing – review & editing (equal). **Howard L. Sofen**: Conceptualization (equal); data curation (equal); formal analysis (equal); investigation (equal); methodology (equal); writing – review & editing (equal). **Angela Y. Moore**: Conceptualization (equal); data curation (equal); formal analysis (equal); investigation (equal); methodology (equal); writing – review & editing (equal). **Mingyue Wang**: Data curation (equal); formal analysis (equal); validation (lead); writing – review & editing (equal). **Deanna Kornacki**: Conceptualization (equal); data curation (equal); formal analysis (equal); methodology (equal); writing – review & editing (equal). **Kathleen Butler**: Conceptualization (equal); data curation (equal); formal analysis (equal); methodology (equal); writing – review & editing (equal). **David Rosmarin**: Conceptualization (equal); data curation (equal); formal analysis (equal); investigation (equal); methodology (equal); writing – review & editing (equal).

## FUNDING INFORMATION

This study was funded by Incyte Corporation (Wilmington, DE, USA).

## Data Availability

Incyte Corporation (Wilmington, DE, USA) is committed to data sharing that advances science and medicine while protecting patient privacy. Qualified external scientific researchers may request anonymised datasets owned by Incyte for the purpose of conducting legitimate scientific research. Researchers may request anonymised datasets from any interventional study (except Phase 1 studies) for which the product and indication have been approved on or after 1 January 2020 in at least one major market (e.g. US, EU, JPN). Data will be available for request after the primary publication or 2 years after the study has ended. Information on Incyte's clinical trial data sharing policy and instructions for submitting clinical trial data requests are available at: https://www.incyte.com/Portals/0/Assets/Compliance%20and%20Transparency/clinical‐trial‐data‐sharing.pdf?ver=2020‐05‐21‐132838‐960.
